# High Power Electromagnetic Waves Exposure of Healthy and Tumor Bearing Mice: Assessment of Effects on Mice Growth, Behavior, Tumor Growth, and Vessel Permeabilization

**DOI:** 10.3390/ijms22168516

**Published:** 2021-08-07

**Authors:** Jelena Kolosnjaj-Tabi, Muriel Golzio, Elisabeth Bellard, Alexandre Catrain, Thomas Chretiennot, Quentin Saurin, Jacques Tarayre, René Vezinet, Marie-Pierre Rols

**Affiliations:** 1 Institut de Pharmacologie et de Biologie Structurale, IPBS, Université de Toulouse, CNRS, UPS, 205 Route de Narbonne, 31400 Toulouse, France; Jelena.Kolosnjaj-tabi@ipbs.fr (J.K.-T.); Muriel.Golzio@ipbs.fr (M.G.); Elisabeth.Bellard@ipbs.fr (E.B.); 2 CEA, DAM, GRAMAT, 46500 Gramat, France; alexandre.catrain@cea.fr (A.C.); thomas.chretiennot@gmail.com (T.C.); quentin.saurin@cea.fr (Q.S.); jacques.tarayre@cea.fr (J.T.); revezat@gmail.com (R.V.)

**Keywords:** high power electromagnetic radiation, electromagnetic environments, in vivo, tumor growth, vessel permeability

## Abstract

High power radiofrequencies may transiently or permanently disrupt the functioning of electronic devices, but their effect on living systems remains unknown. With the aim to evaluate the safety and biological effects of narrow-band and wide-band high-power electromagnetic (HPEM) waves, we studied their effects upon exposure of healthy and tumor-bearing mice. In field experiments, the exposure to 1.5 GHz narrow-band electromagnetic fields with the incident amplitude peak value level in the range of 40 kV/m and 150 MHz wide-band electric fields with the amplitude peak value in the range of 200 kV/m, did not alter healthy and tumor-bearing animals’ growth, nor it had any impact on cutaneous murine tumors’ growth. While we did not observe any noticeable behavioral changes in mice during the exposure to narrow-band signals when wide-band HPEM signals were applied, mice could behave in a similar way as they respond to loud noise signals: namely, if a mouse was exploring the cage prior to signal application, it returned to companion mates when wide-band HPEM signals were applied. Moreover, the effect of wide-band signals was assessed on normal blood vessels permeability in real-time in dorsal-chamber-bearing mice exposed in a pilot study using wide-band signal applicators. Our pilot study conducted within the applicator and performed at the laboratory scale suggests that the exposure to wide-band signals with the amplitude of 47.5 kV/m does not result in increased vessel permeability.

## 1. Introduction

Electromagnetic environments, such as the ones generated by high-power microwaves and high-power radiofrequencies, might be used to disrupt the functioning of electronic devices transiently or permanently. As members of military personnel or civilians might potentially be exposed to such signals, the biological effects of these signals should be evaluated to determine the biological effects of such exposures.

As electromagnetic fields are implicated in a series of biochemical processes [1,2], exposure to external electromagnetic fields, and namely the high power electromagnetic (HPEM) fields [3], could have an impact on certain physiological functions. While short-term thermal effects may indeed occur after radiofrequency radiation exposure, their effect is relatively well known [4] and served to establish the ICNIRP guidelines [5]. Conversely, the occurrence of short- and long-term non-thermal effects, including a potential cell membrane permeabilization, increased carcinogenesis, or increased vascular permeability, is still poorly addressed and controversial.

In this context, we have previously evaluated the effects of high-power electromagnetic signals on different biological models of increasing complexity [6,7]. Among them, we used giant unilamellar vesicles, which are the simplest model, mimicking the cell membrane; normal cells (fibroblasts, isolated from a healthy skin biopsy) as well as cancer cells (human colon carcinoma HCT-116 cell line) [6]; and multicellular spheroids made of fibroblasts or cancer cells [7], which are a model mimicking small avascular tumors [8].

In our previous studies, the exposure to high-power electromagnetic signals, such as the ones generated by a commercial DIEHL DST110T high-power system (signals applied at a frequency of 200 MHz with 200 kV/m amplitude radiated waves, pulse duration of 20 ns, and 2500 delivered pulses) and signals such as the ones generated by the GERAC TEMPETE high-power system (applied at a 1.5 GHz frequency with 40 kV/M amplitude, pulse duration of 4 µs, and 50,000 pulses) did not result in giant unilamellar vesicles deformation nor in cell membrane permeabilization [6]. Moreover, exposures of multicellular spheroids to such high-power electromagnetic signals did not affect the macroscopic aspect, spheroids’ growth, cell membrane integrity, ATP content, and mitochondrial potential. In addition, signal exposure did not induce apoptosis of cells constituting the spheroids [7].

In mentioned experiments [6,7], the electromagnetic exposure did not induce any measurable detrimental effects. Evidently, not observing an effect following a given number of biochemical tests performed in vitro does not allow us to ascertain that there are no biological effects at all. Therefore, additional, namely, in vivo studies, were deemed necessary to further evaluate the potential impact of high-power electromagnetic (HPEM) signals.

Animal body weight is an important and extremely sensitive measurable variable, which excellently reflects nuisance induced by prospective stressors. Decreased animal growth is thus regarded as a pillar variable in both acute and chronic toxicity studies. As a further step to experiments performed on giant unilamellar vesicles and living cells [6,7], the aim of this study was to evaluate the effects of HPEM signals—narrow-band and wide-band high-power electromagnetic waves—on living animals. In this context, we studied the exposure of healthy and tumor-bearing mice. 

As there are little scientific data involving co-exposure scenarios, including concomitant exposure to electromagnetic signals and cancer, melanoma cell-based cutaneous tumor-bearing mice were also exposed to HPEM signals.

In our field experiments setting, animal and tumor growth was thus monitored over time. 

Electromagnetic radiation is composed of the magnetic and electric field components, and intense electric field pulses are known to induce the permeabilization of cell membranes and vessels, which our group intensively studied for decades. Therefore, to assess if vessels in healthy mice become permeabilized upon HPEM exposure, we herein also focused on normal vessels permeabilization visualization during HPEM exposure. For this purpose, a pilot study was performed, where we tested an HPEM signals applicator, which allowed real-time vessel visualization during animals’ exposure in laboratory tests. 

**Figure 1:**
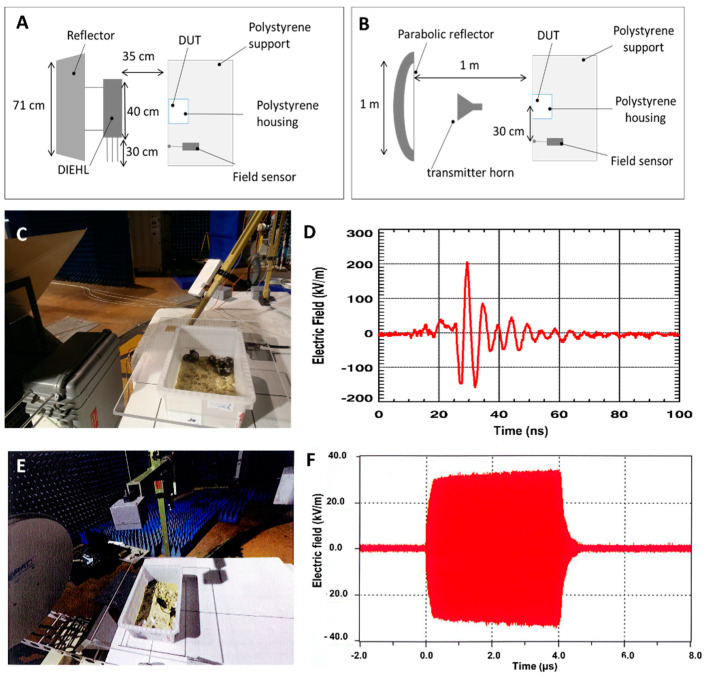
High−power electromagnetic (HPEM) exposure experimental setting in field experiments. (**A**) Schematic representation of the wide−band exposure set up. (**B**) Schematic representation of the narrow−band exposure set up. (**C**) Photograph of the animals exposed to wide−band signals. (**D**) Measured incident electric fields of wide−band signals. (**E**) Photograph of the animals exposed to narrow−band signals. (**F**) Measured incident electric fields of narrow−band signals.

In summary, in one set of experiments, healthy and tumor-bearing mice were exposed to HPEM signals generated by high-power antennas in field experiments (Figure 1), to see if and to what extent HPEM signals affect healthy and tumor-bearing animals growth and tumor growth.

**Figure 2:**
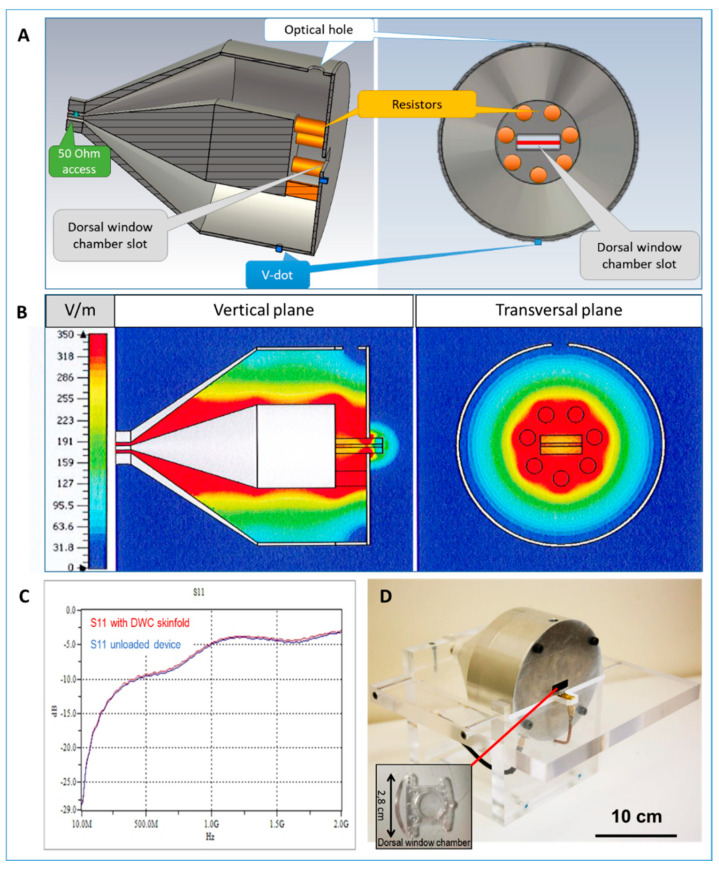
Design and properties of the coaxial dorsal window chamber (DWC) applicator. (**A**) Schematic representation of the longitudinal and the cross section of the applicator. (**B**) The E−field homogeneity as evaluated by 3D electromagnetic simulation using 3D CST software calculated over the vertical (left) and the transversal plane (central section of the test volume) of the applicator loaded with the murine skinfold. (**C**) Measured S11 parameter of the applicator (loaded and unloaded with the DWC). (**D**) Photograph of the applicator and the DWC (inset figure), which is inserted into the slot (red line) and allows vasculature monitoring under the microscope (via the optical hole at the top of the device). The inset shows an empty DWC. The figure is adapted from reference [9].

In the other set of experiments, in a pilot study aiming to test immediate effects during animals’ exposure to HPEM signals, mice were exposed at the laboratory scale using a specific HPEM signal applicator (Figure 2). In order to assess the effects of HPEM on vasculature permeabilization, healthy mice underwent dorsal window chambers (DWC) surgery. Skinfolds in DWCs were subsequently exposed to signals generated within the HPEM applicator (Figure 2), where we could observe if permeabilization occurs following HPEM signal exposure. This setting was tested in a pilot study to roughly apprize the effect of HPEM signals on healthy vasculature’s permeability.

## 2. Results

The effects of HPEM exposure were evaluated in field experiments and at a laboratory scale in a dedicated applicator (Figure 1 and Figure 2). Signal exposure protocols are summarized in Table 1.

### 2.1. Exposure to Narrow-Band Signals Generated in Field Experiments

**Figure 3:**
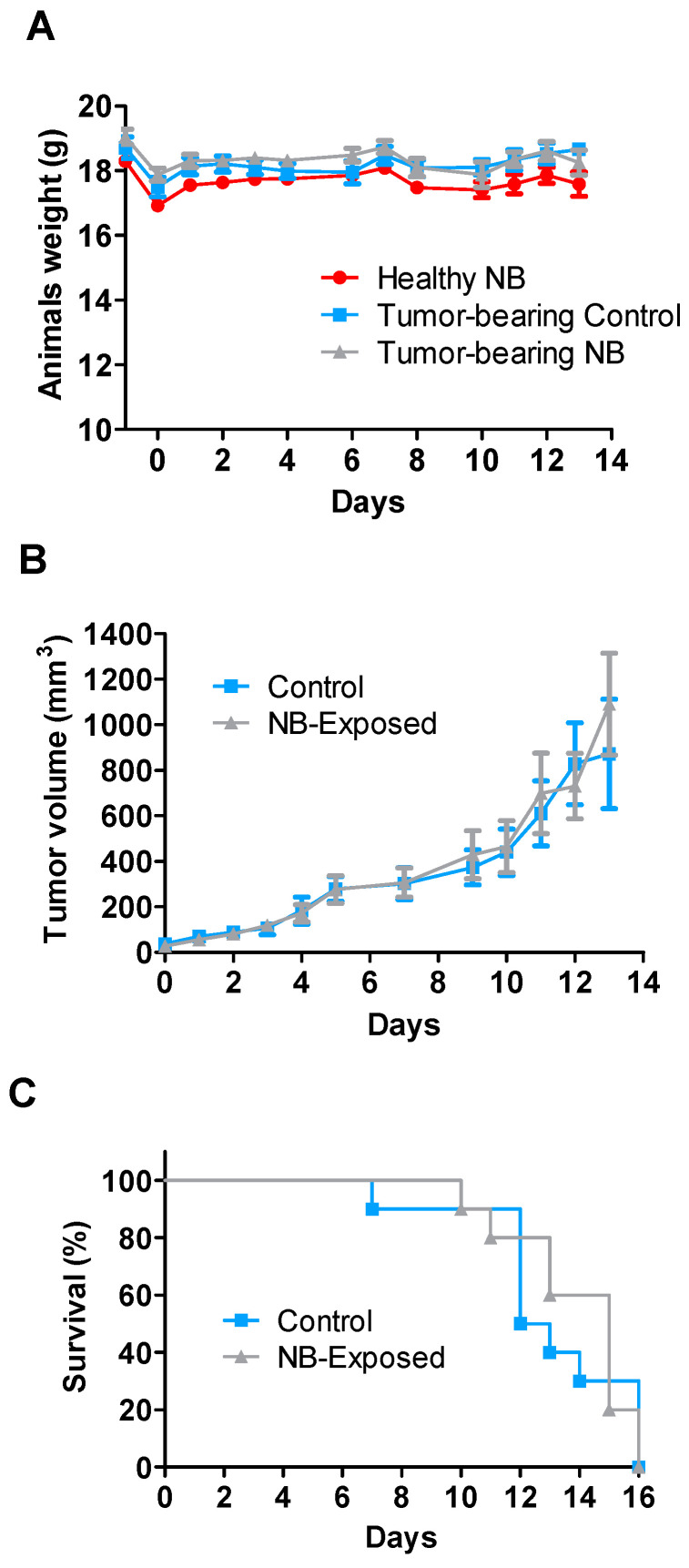
(**A**) Animal growth curves, (**B**) Tumor growth curves, and (**C**) Tumor−bearing animals’ survival plot after animals exposure to narrow−band (NB) signals generated by the TEMPETE narrow-band signal generating system (N = 10 mice/group). *Ad* (**A**) Healthy animals were weighted two times on the day of HPEM exposure (D0): before and after arriving in the hangar, where field experiments were performed. *Ad* (**B**) Tumor−bearing animals were exposed 7 days following cancer cells inoculation and were sacrificed when the tumor volume was beyond the limit authorized by the ethics committee.

All figures and tables should be cited in the main text as Figure 1, Table 1, etc. The exposure to HPEM signals generated by the narrow-band TEMPETE system, delivering signals described in Table 2, does not affect animal behavior and animal growth. Moreover, signal exposure does not affect the growth of tumors in tumor-bearing mice. The results of animal and tumor growth are summarized in Figure 3. A representative video showing mice during signal exposure can be found in supporting information (Video S1).

### 2.2. Exposure to Wide-Band Signals Generated in Field Experiments

**Figure 4:**
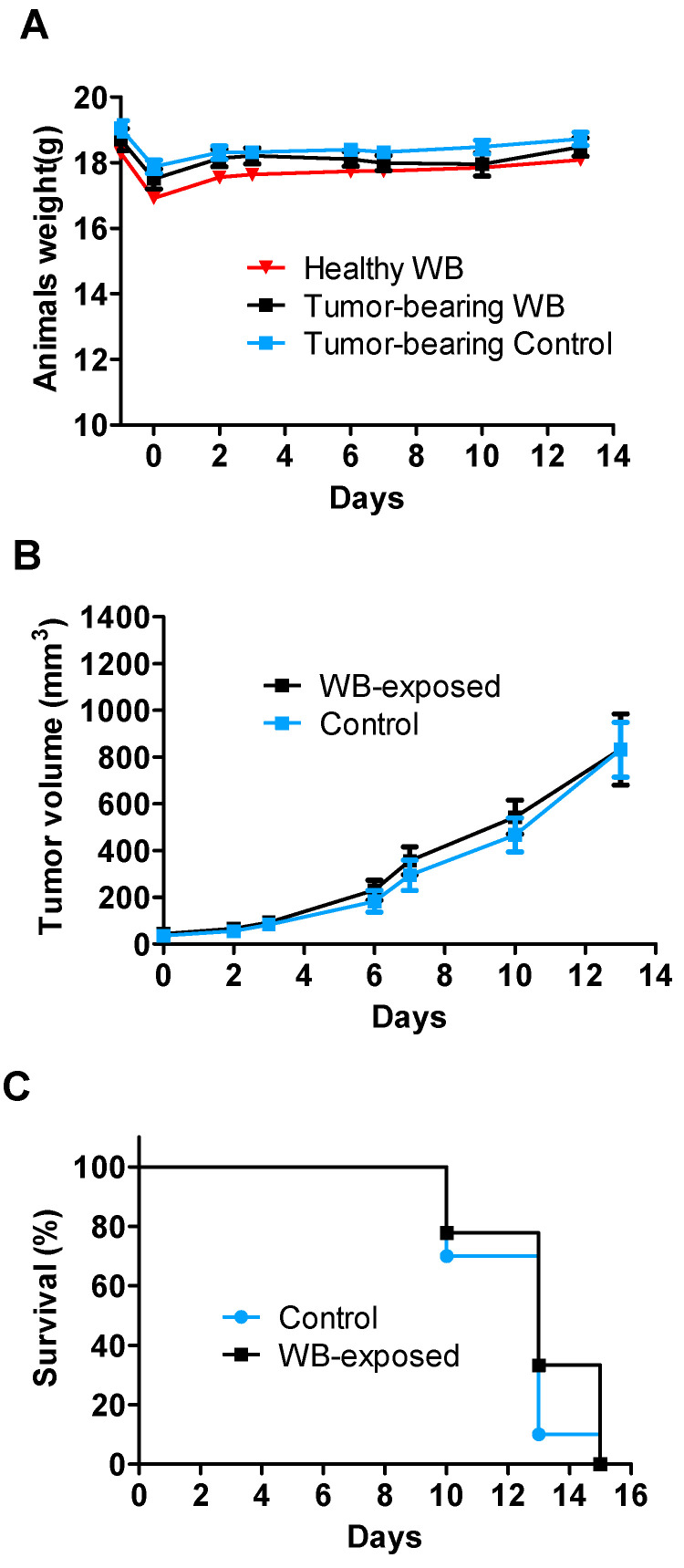
(**A**) Animal and (**B**) Tumor growth curves and (**C**) Tumor−bearing animals survival plot after animals’ exposure to wide−band signals generated by the DIEHL DS 110 wide−band (WB) signal generating system (N = 10 mice/group). *Ad* (**A**) Healthy animals were weighted two times on the day of HPEM exposure (D0): before and after arriving to the hangar, where field experiments were performed. *Ad* (**B**) Tumor−bearing animals were exposed 7 days following cancer cells inoculation and were sacrificed when the tumor volume was beyond the limit authorized by the ethics committee.

The exposure to HPEM signals generated by the wide-band DIEHL DS 110 system, delivering signals described in Table 3, does not affect animal growth, as well as it does not affect the growth of tumors in tumor-bearing mice. The results of animal and tumor growth are summarized in Figure 4. Nevertheless, during wide-band signal exposure, we note that the behavior of one animal is similar to the behavior that occurs when the animals are exposed to a loud siren-like noise (Supporting Information Video S2). Precisely, the animal, which left the grouped companion mice to explore the cage in the absence of the signals, returns to its mates during signal application.

### 2.3. Animal Exposure to Wide-Band HPEM Signals Generated at the Laboratory Scale and Animals Exposure to Vessel-Permeabilizing Electric Field (Positive Control) 

**Figure 5:**
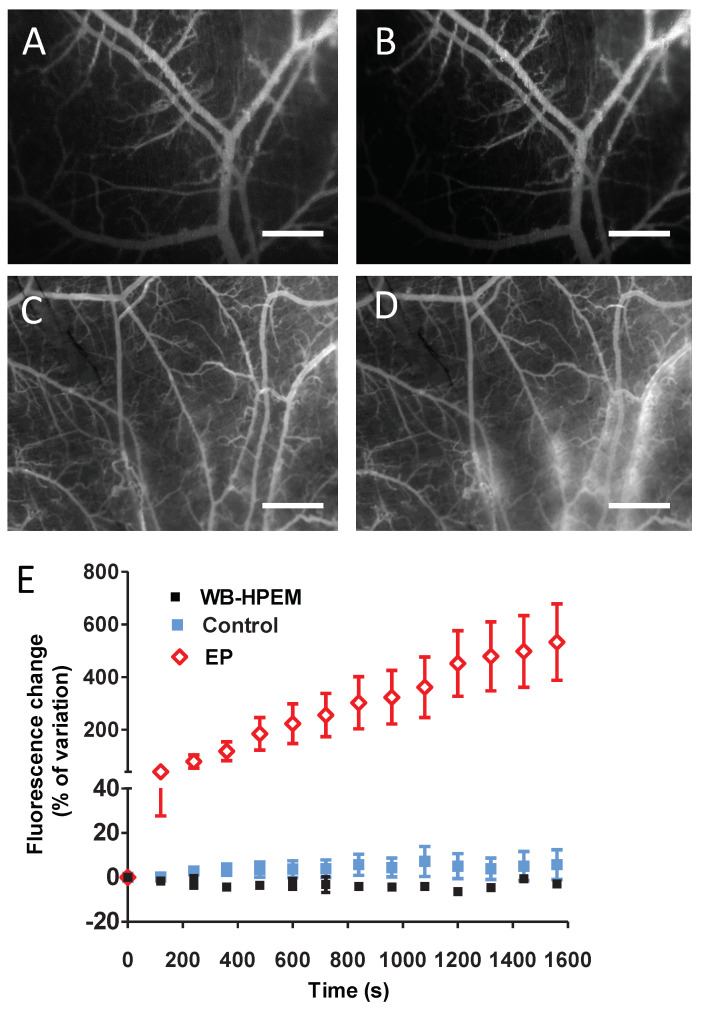
Vessel permeability assessment by fluorescence microscopy after intravenous injection of FITC−dextran (70 kDa) to healthy mice. Micrographs taken prior to (**A**) and after (**B**) skinfolds exposure to wide−band (WB) signals generated within the dedicated HPEM applicator. Micrographs taken from the positive control, prior to (**C**) and (**D**) after application of pulsed electric field, which has a recognized vessel permeabilizing action. (**E**) Relative mean fluorescence intensity measurements outside the vessels as a function of time during and after illumination (N = 3 mice/group).

The potential permeabilization effect, induced by wide-band signals generated within the HPEM applicator, was assessed in healthy dorsal window chamber (DWC)-bearing mice. Vessel permeabilization was assessed by real-time fluorescence microscopy of the vasculature in the murine skinfold-containing DWCs, following an IV injection of 100 μL of FITC-dextran (70 kDa) (Figure 5A–D)). Four consecutive illuminations were applied. The parameters applied during the exposure protocol are summarized in Table 4, and the signal bursts are represented with arrows in Figure 5E. No variation of the relative mean fluorescence intensity as a function of time was observed outside the vessels when compared to non-illuminated samples (Figure 5E). Conversely, dextran leakage is, as expected, observed in the positive control, when permeabilizing electric field (eight square electric pulses, field strength 1300 V/cm, pulse length 100 μs, repetition frequency 1 Hz) is applied, according to the procedure that was previously described [10,11].

## 3. Discussion

The assessment of biological effects induced by HPEM signals is pivotal for the determination of potential health risks, to which at this stage, the personnel using such generators in experimental settings, and prospectively, the personnel of the armed forces as well as potentially the civilians, might be exposed. 

Electromagnetic fields can act on biological tissues by thermal and non-thermal mechanisms [12]. As limited exposure time to high-power electromagnetic environments is sufficient to disrupt the functioning of electronic devices, the HPEM exposures are generally of short duration and thus have a low radiated electromagnetic energy, which does not cause significant tissue heating. Among evaluated high-power electromagnetic signals, we have previously reported that wide-band (also termed meso-band signals) induced no detectable temperature increase in exposed samples (namely, in cell growing media in which biological cells and multicellular spheroids were exposed) [7]. Thus, under such circumstances, electromagnetic fields may rather induce cellular effects by non-thermal mechanisms. In our previous study, we evaluated the effect of wide-band and narrow-band HPEM signals on normal dermal fibroblasts and cancer cells (HCT-116) spheroids, where we assessed cells growth, plasma membrane integrity, induction of apoptosis, ATP content, and mitochondrial potential. Under our experimental conditions, the exposure to wide-band and narrow-band HPEM signals did not induce any significant alteration of assessed parameters [7].

In the continuity of the assessment of biological effects upon HPEM exposures, in this study, we evaluated the exposure of healthy or tumor-bearing animals to wide-band and narrow-band signals, with field strengths of 200 kV/m and 40 kV/m, respectively, generated by the TEMPETE or DIEHL DS 110 system. 

In our previous studies [6,7], we studied the effects of HPEM waves on giant unilamellar vesicles, colorectal cancer cells, and normal dermal fibroblasts cultured in 2 and 3D. In these models, we observed no effects in vitro for the given battery of performed tests. Indeed, while we performed a given number of cellular tests (that we considered the most relevant), other unscreened effects might have happened. To see if HPEM might ail animals, we conducted this in vivo study.

In the present study, we assessed the effect of HPEM on healthy mice at first, and subsequently, we chose a melanoma model to see if HPEM signals might aggravate cancer (e.g., amplify the speed of tumor growth). The used melanoma cutaneous tumor model was considered relevant, as the skin acts as the first barrier facing radiation exposure.

In our experimental setting, HPEM exposure does affect neither animals’ growth nor tumor growth. However, while no behavioral changes were detected in mice during the application of narrow-band signals, when wide-band HPEM signals were applied, mice could behave in a similar way as they respond to noise signals. The hypothesis of the occurrence of an auditory effect was raised because previous findings highlighted that pulsed HPEM signals induce the so-called microwave auditory effect or Frey effect, which was first described in 1961 by the American neuroscientist Allan H. Frey [13]. This effect is due to short-lived and low-scale heating (in the range of 10.5 °C–10.6 °C), which occurs in the head due to E-field exposure [14,15,16]. The hearing of microwave pulses involves electromagnetic waves with frequency ranges from tens of MHz to tens of GHz [15,16], which cause a mechanical shock due to thermo-elastic expansion. The thermo-elastic wave induces acoustic pressure, which travels by bone conduction to the inner ear, and activates the cochlear receptors via the same process as the one that is involved in normal hearing. Overall, this auditory effect results in the perception of clicking or knocking noises and can sometimes even be perceived as speech. While at the current stage, we do not have irrefutable proof of whether this auditory phenomenon actually occurred in exposed mice, it might be considered as a potential secondary effect associated with RF-induced thermal action. In any event, the eventual occurrence of this phenomenon has no evident detrimental health effects [16], and did not, in our case, correlate neither to animal weight loss nor did it affect tumor growth.

In addition to field experiments, we also conducted laboratory experiments in order to assess potential vessel permeabilization in exposed animals. Intense electric field pulses may increase the permeability of blood vessels [10,17]. While this effect has not been ascertained in vivo [18], it has been suggested that 1.8 GHz GSM-like electromagnetic fields might increase the permeability of the blood–brain barrier in in vitro models [19]. 

We, therefore, sought to assess if HPEM signals might potentially contribute to vessel permeabilization. In a pilot study, which aimed to assess prospective acute effects of HPEM signals in vivo and in real-time, we have created a dedicated EM applicator and sought to visualize potential effects of wide-band signals on murine vasculature with HPEM signals delivered at the laboratory scale. In our laboratory experiments, performed at peak E-field values of 47.5 kV/m, we did not observe any increase in blood vessels permeability. The Incident E-field value, representing the maximum absolute zero to peak value obtained within the applicator applied in this study, equaled 47.5 kV/m and was obtained with a voltage generator supplying 950 V. Indeed, the peak value obtained in field experiments by the DIEHL DS 110 system was higher, as it equaled 200 kV/m. In order to reproduce these values at a laboratory scale, we would require a voltage generator supplying 4 kV. The development of such a generator is theoretically possible, yet it implies substantial technical challenges, and we are currently working on this project. Alternatively, a different solution would imply the use of a power amplifier, which would be able to deliver a prohibitive peak power of 320 kW. While the E-field values in laboratory experiments did not equal the E-field in field experiments, our experiments allowed the monitoring of acute biological effects upon HPEM exposure at the microscopic scale and in real-time. In addition, these monitoring methods could be applied to follow up reiterated/chronic exposures of laboratory animals.

## 4. Materials and Methods

### 4.1. In Vivo Studies

Animal studies and the expertize involved in animal experiments was provided by the IPBS staff. In vivo studies were performed on adult (8 weeks old) female C57BL6 mice (Charles River Laboratories, Saint Germain Nuelles, France) weighing between 21 and 26 g. Animal experiments were conducted in accordance with French procedural guidelines for animal handling and obtained approval from the National Ethical Review Committee (2015032613444287). Sixty-nine mice were used in the study (the numbers of animals per group are summarized in Table 1). The animals were housed at 20 °C and allowed to acclimate to the facility for at least 1 week prior to being injected with tumor cells or operated on (implanted with dorsal window chambers).

#### 4.1.1. Subcutaneous Tumor Xenografts

Thirty mice were injected with murine melanoma cells (B16-F10, ATCC^®^ CRL-6475™, ATCC, Manassas, VA, USA). Each mouse was injected with 1 × 10^6^ tumor cells suspended in 100 µL of PBS. The mice were considered ready for treatment 7 days after tumor cell injection when the tumors reached approximately 30 mm^3^.

#### 4.1.2. Dorsal Window Chambers (DWC) Surgery

Nine mice underwent DWC surgery. The procedure was performed as previously described [10]. Briefly, surgery was carried out under general anesthesia using intraperitoneal injection of Ketamine (1 mg/mL, MERIAL, Lyon, France) and Xylazine (5 mg/mL, Bayer, Puteaux, France). Non-metallic dorsal window chamber kits were used (APJ Trading Co., Ventura, CA, USA). Two plastic frames were sandwiched on an extended double layer of skin with the use of nylon screws and sutures. Before the implantation of the frames, a circular hole with a diameter of approximately 12 mm was made in one of the skin layers. The superficial tissue was surgically removed, leaving a facial plane with associated vasculature. Ensuing the surgery and the following day, a non-steroidal anti-inflammatory agent (Profenid, Sanofi-Aventis, Paris, France) was injected intramuscularly (10 mg/kg, 50 μL in each thigh), to provide analgesia and to avoid inflammation [10,17].

#### 4.1.3. Animal and Tumor Growth

Animals were weighed on a daily basis. On the day of the transfer to the facility where field experiments were performed, the animals were weighed twice per day: prior to departure to the hangar and upon arrival from the hangar. The tumors were measured with a digital caliper every day, and the volumes were calculated from the formula V = Dd^2^/2, where D is the longest diameter and d is the diameter perpendicular to D. The study was discontinued at D20 when most tumors attained 1000 mm^3^, which was considered the ethically acceptable tumor volume limit.

### 4.2. Electromagnetic Exposure

The electromagnetic exposure means (generator, applicators) and metrology were provided by the CEA-Gramat.

#### 4.2.1. Exposure to HPEM Signals Generated by High-Power Antennas

Mice were transferred to the hangar on the day of the illumination experiment. The transport time lasted approximately 2 hours. The animals were transported and exposed to HPEM waves in the Small Rodent Shipping Container (Ref. No Z683191, Sigma-Aldrich, Saint Quentin Fallavier, France). During their stay in the facility (prior and after illumination), the animals were housed at 20 °C. The cage (45 cm × 29 cm × 19 cm) containing 5 animals was placed on polystyrene support and placed 1.5 m above the ground at 35 cm or 1 m distance from the center of the emission axis of the antenna (Figure 1A,B). An electric field sensor was systematically placed in the vicinity of the device under test-DUT (the animals containing cage) at an equal distance as the one between the antenna and the DUT, either in the same axis or at a position that was laterally shifted for 30 cm. The schematic representation of this kind of set-up is shown in Figure 1A,B. The experimental settings and the corresponding electric fields measured by the field sensor are represented in Figure 1C–F. The electric fields within the DUT can thus be derived from the values of the incident electric fields measured by the sensor (as the field at the location of the DUT and the field at the location of the field sensor have a constant-coefficient difference). Electromagnetic signals were generated by the narrow-band (NB) TEMPETE high-power system or by the Wide-band (WB) system DIEHL DS 110. The electromagnetic parameters applied during NB illumination and WB illumination procedures are described in Table 2 and Table 3, respectively. When the animals were placed at the site of signal exposure (Figure 1C,E) prior to exposure to HPEM signal bursts, the animals were exposed to a noise signal (Supporting Information Videos S1 and S2). This approach was used to allow discrimination of animals’ behavior response to noise from their potential response to HPEM signal exposure. The cage of the control mice was placed in a Faraday cage during illumination protocols. The mice were brought back to the animal housing facility of the research laboratory on the same day.

#### 4.2.2. Exposure to HPEM Signals Generated within the Dedicated Applicator

Electromagnetic signals (Table 4), corresponding to electromagnetic waves generated by the Wide-band system DIEHL DS 110, were generated by a dedicated broad-band high-pulsed power coaxial applicator specifically designed for in vivo biological experiments, Ref. [13] coupled to a homemade generator based on high-voltage MOSFET and resonant transmission line, providing a 150 MHz damped sinusoid signal with a 1 kV amplitude.

The technical specifications of the applicator, namely the applicator’s design, S11 parameter, and the numerical estimations of the homogeneity of the electric field (estimated to 85% within the volume of interest), were described previously [9] and are briefly summarized in Figure 2. To sum up [9], the coaxial applicator (Figure 2) is characterized by a circular array of resistors (Figure 2A), creating a homogenous electric field within the internal volume, delimitated by the array of resistors, allowing the application of high power pulsed electric fields to DWCs containing skinfolds of healthy living mice. Figure 2B shows the maxima of the calculated E-field across the test volume, and Figure 3C shows the measured S11 parameter of the loaded and unloaded applicator. The DWCs are inserted into the slot in the middle of the extremity plate (Figure 2D). A lateral hole within the external conductor (named optical hole) (Figure 2A) enables real-time microscope observations of the murine skinfold. [9]

The illuminations of skinfolds were made 2 days following DWC installation surgery. Mice, on which no illumination was applied, served as the control group. Fluorescein isothiocyanate (FITC) dextrans (70 kDa) (Sigma Chemical Co. (St. Louis, MO, USA)) were dissolved in phosphate-buffered saline (PBS) and washed through 30 kDa spin filters (Sartorius Stedim Biotech GmbH, Goettingen, Germany) and centrifuged at 1000× *g* to remove any free fluorophores or low molecular weight compounds. The high molecular weight component was resuspended in PBS at 37.5 mg/mL and used in mice at 3.75 mg/100 μL. Fluorescent dextrans (FDs) were injected in the retro-orbital plexus before illumination to observe any extravasation of FDs into the tissue [10].

#### 4.2.3. Exposure to Vessel-Permeabilizing Electric Field Pulses

The DWC-bearing mice exposed to the permeabilizing electric field were used as positive controls [10]. Vessel permeabilization occurred after application of eight square monopolar electric pulses, with a field strength of 1300 V/cm, pulse length 100 μs, and repetition frequency of 1 Hz, generated by an electric pulse generator S20 (Leroy Biotech, Saint-Orens-de-Dameville, France). The pulses were applied as described previously [10,11], with two parallel stainless-steel electrodes (length 5 mm, width 1.3 mm) 4 mm apart, which were placed in contact with the skinfold-containing DWC, on which conductive gel (Eko-gel, Egna, Italy) was spread, to ensure signal transmission between the electrodes.

### 4.3. Intravital Imaging

Intravital fluorescence macroscopic imaging (“macroscopy”) was performed on anesthetized animals. The “Macrofluo” fluorescence microscope (Leica Microsystems SA, Rueil-Malmaison, France), equipped with a Cool Snap HQ2 Camera (Roper Scientific, Photometrics, Tucson, AZ, USA), was used as previously described [10]. After IV injection of 100 μL of FDs, vessels were imaged by fluorescence using appropriate filters (excitation filter, BP: 480/40 nm, emission filter, BP 527/30 nm). Sequences of images were acquired covering a 30 min period before, during, and after signal exposure. The files were stored and analyzed offline after image acquisition (Metavue, Metamorph, Molecular Devices, Sunnyvale, CA, USA and software ImageJ, National Institute of Mental Health, Bethesda, MD, USA). The fluorescence intensity outside the blood vessels was determined after image processing as previously described [10,11]. After data processing, variations in fluorescence intensity outside the vessels were obtained.

### 4.4. Statistical Analyses

Ten mice per group were used in field experiments (where healthy or tumor-bearing mice were submitted to signals generated by antennas), and 3 mice per group were used in the experiment at the laboratory scale (submitted to signals generated within the applicator). Data are expressed as mean ± SEM, and overall statistical significance was set at *p* ≤ 0.005.

## 5. Conclusions

The primary aim of our study was to assess the impact of HPEM radiation on living animals, to discern if there is any exposure-related risk to humans, particularly to persons who are occupationally exposed to such signals. The exposures to 1.5 GHz narrow-band electromagnetic fields with the incident amplitude level of 40 kV/m, and 150 MHz wide-band electric fields with the amplitude in the range of 200 kV/m, do not induce any significant alterations of animal or tumor growth. At first, our aim was to assess if HPEM exposure alters the growth of healthy animals, as animal weight gain is among the most sensitive variables that reflect animal wellbeing, and animal growth follow-up is among the pillar parameters that allows establishing any potential harm. In our setting, animal growth was not affected following HPEM signal exposure. 

As in real life, we are rarely confronted with one single stressor, and as cancer, at a certain point of life, affects one person out of two, the second question was how tumors, and particularly, cutaneous tumors made by intradermal inoculation of melanoma cells, might respond to HPEM exposure. In our setting cutaneous tumors growth was not affected following a single exposure to HPEM described signals.

Nevertheless, our study indicates that when wide-band HPEM signals are applied, mice may behave in a similar way as they respond to loud noise signals. 

Moreover, as assessed in a pilot study on a small number of mice, the exposure to wide-band signals with the amplitude of 47.5 kV/m does not induce vessel permeabilization, as assessed in real-time in healthy dorsal-chamber-bearing mice exposed using wide-band signal applicators.

## Figures and Tables

**Table 1:** Signal exposure protocols applied to healthy (C57BL6), tumor-bearing (C57BL6 + B16-F10), and dorsal chamber-bearing (C57BL6 + DWC) mice.

	Number of Animals Used in Field Experiments (Signals Generated by Antennas)			Number of Animals Used in Laboratory Experiments (Signals Generated in Applicators)			
	Narrow-Band	Wide-Band	Sham	Narrow-Band	Wide-Band	Sham	Positive Control
C57BL6	10	10	10	-	-	-	-
C57BL6 + B16-F10	10	10	10	-	-	-	-
C57BL6 + DWC	-	-	-	- (*)	3	3	3

* Mice were not exposed to narrow-band signals in laboratory experiments due to technical limitations related to the generator power. For further information, please refer to the Discussion.

**Table 2:** Electromagnetic parameters applied in field experiments for narrow-band (NB) illuminations of healthy and tumor-bearing mice (N = 10 mice/group).

	Expected E-Field	Measured Mean Incident E-Field	Width	Number of Pulses	Number of Bursts	Delay between Bursts	Signal Frequency	PRF
Sham (healthy mice)	0	0	0	0	0	0	0	0
Sham (tumor-bearing mice)	0	0	0	0	0	0	0	0
Illuminated (healthy mice)	40 kV/m	34.55 kV/m	4 µs	5000	20	15 s	1.5 GHz	500 Hz
Illuminated (tumor-bearing mice)	40 kV/m	36.55 kV/m	4 µs	5000	20	15 s	1.5 GHz	500 Hz

**Table 3:** Electromagnetic parameters applied in field experiments for wide-band (WB) illuminations of healthy and tumor-bearing mice (N = 10 mice/group).

	Expected E-Field	Measured Mean Incident E-Field	Width	Number of Pulses	Number of Bursts	Delay between Bursts	Signal Frequency	PRF
Sham (healthy mice)	0	0	0	0	0	0	0	0
Sham (tumor-bearing mice)	0	0	0	0	0	0	0	0
Illuminated (healthy mice)	190 kV/m	199 kV/m	4 µs	5 00	5	115 s	195 MHz	100 Hz
Illuminated (tumor-bearing mice)	190 kV/m	203 kV/m	4 µs	5 00	5	115 s	195 MHz	100 Hz

**Table 4:** Electromagnetic parameters applied in laboratory experiments for wide-band (WB) illuminations of dorsal window chamber (DWC)-bearing mice.

	E-Field	Width	Number of Pulses	Number of Bursts	Delay between Bursts	Signal Frequency	PRF
Sham DWC-bearing mice	0	0	0	0	0	0	0
Illuminated DWC-bearing mice	47.5 * kV/m	5 ns	2500	4	120 s	200 MHz	100 Hz

* The E-field value is below the ones radiated by the Diehl system; please refer to the discussion section where the underlying technical background is explained in detail.

## Supplementary Materials

The following are available online at https://www.mdpi.com/article/10.3390/ijms22168516/s1.
