# Supplementary material for: High Power Electromagnetic Waves Exposure of Healthy and Tumor Bearing Mice: Assessment of Effects on Mice Growth, Behavior, Tumor Growth, and Vessel Permeabilization
Source: Int J Mol Sci. 2021 Aug 7;22(16):8516. doi: 10.3390/ijms22168516 (PMC8395230; doi:10.3390/ijms22168516)
Supplement: Supplementary file 1 [file ijms-22-08516-s001.zip › ijms-1316991-supplementary.pdf]

Please refer to 10.5281/zenodo.5092170
